# Roles of PFKFB3 in cancer

**DOI:** 10.1038/sigtrans.2017.44

**Published:** 2017-11-24

**Authors:** Linlin Shi, Hongming Pan, Zhen Liu, Jiansheng Xie, Weidong Han

**Affiliations:** 1 Department of Medical Oncology, Sir Run Run Shaw Hospital, College of Medicine, Zhejiang University, Hangzhou, Zhejiang, China; 2 Laboratory of Cancer Biology, Institute of Clinical Science, Sir Run Run Shaw Hospital, College of Medicine, Zhejiang University, Hangzhou, Zhejiang, China

## Abstract

The understanding of 6-phosphofructo-2-kinase/fructose-2,6-biphosphatase 3 (PFK-2/FBPase 3, PFKFB3) has advanced considerably since its initial identification in human macrophages in the mid-1990s. As a vital regulator of glycolysis, accumulating studies have suggested that PFKFB3 is associated with many aspects of cancer, including carcinogenesis, cancer cell proliferation, vessel aggressiveness, drug resistance and tumor microenvironment. In this review, we summarize current knowledge of PFKFB3 regulation by several signal pathways and its function in cancer development in different cell types in cancer tissues. Ubiquitous PFKFB3 has emerged as a potential target for anti-neoplastic therapy.

## Introduction

Glycolysis is the metabolic pathway that converts glucose to pyruvate. The free energy released in this process is utilized to form the high-energy compounds ATP and NADH. A high rate of glycolytic flux, even in the presence of oxygen, is a central metabolic hallmark of tumors. This phenomenon is historically known as the ‘Warburg Effect’.^1^ The rate of glycolytic flux is controlled at different levels and by different mechanisms. One of the critical modulators is the conversion of fructose-6-phosphate (F6P) to fructose-1,6-bisphosphate (F1,6P2) by 6-phosphofructo-1-kinase (PFK-1), which is the first committed rate-limiting step of glycolysis.^2^ The intracellular allosteric regulator fructose 2,6-bisphosphate (F2,6P2) is a potent activator of PFK-1.^3^ F2,6P2 increases the affinity of PFK-1 for F6P and overrides the tonic allosteric inhibition of PFK-1 by ATP, allowing glycolytic flux through the PFK-1 checkpoint and into F1,6P2 synthesis.^3^ The intracellular steady-state concentration of F2,6P2 is controlled by a family of homodimeric and bifunctional enzyme PFK-2/FBPase (PFKFB).^4,5^ Despite the high sequence homology (85%) of their core catalytic domains, the four isozymes of PFKFB (PFKFB1–4) display distinct properties, including tissue expression profiles, the ratio of their kinase/phosphatase activities, and their response to protein kinases, hormonal and growth factor signals.^6,7^ The bifunctional isoenzyme encoded by the *pfkfb3* gene has the highest kinase: phosphatase activity ratio, which in turn sustains high glycolytic rates.^8^ Furthermore, PFKFB3 exists different spilce variants. For example, six splice variants of PFKFB3 have been found in the human brain.^9^ Confirming the activity and localization of these splice variants may help improve our understanding of the regulation of PFKFB3 and its function in tumor cell glycolysis, as well as its requirement for tumor growth.

**Figure 1:**
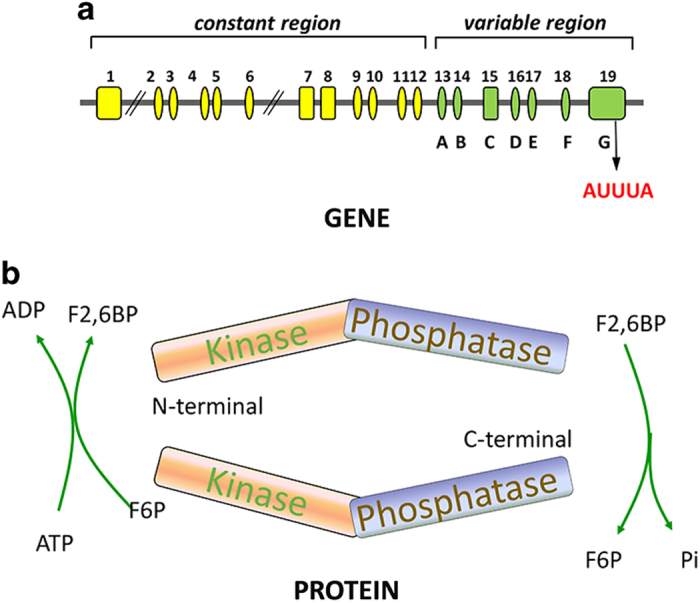
General structure of the *PFKFB3* gene and protein. (**a**) The *PFKFB3* gene contains at least 19 exons, which can be divided into 2 regions, the constant and variable regions. The variable region contains seven exons named A–G, and variations in the exons in this region leads to six isoforms of PFKFB3. PFKFB3 contains multiple copies of the AUUUA instability element in its 3′UTR. (**b**) The PFKFB3 protein has two homodimeric subunits. Each subunit of PFKFB3 comprises two functional domains: an N-terminal kinase domain and a C-terminal phosphatase domain. The kinase activity catalyzes the production of F2,6P2 and ADP from F6P and ATP, which highly promote the glycolytic pathway. The phosphatase activity dephosphorylates F2,6P2 to produce F6P and Pi.

The *pfkfb3* gene is localized on chromosome 10p15.1 (ref. ^10^) and contains multiple copies of the oncogene-like AUUUA instability element in its 3ʹ untranslated region (3′UTR) (Figure 1a).^11^ The *pfkfb3* gene contains at least 19 exons, and alternative splicing of the COOH-terminal variable region leads to the expression of at least six structural isoforms, termed UBI2K1–6 in humans^9^ (Table 1). The PFKFB3 protein consists of two homodimers. The monomer structure is divided into two functional domains within the same polypeptide chain.^4,7,12^ The C-terminal domain contains the bisphosphatase activity of the enzyme.^13–15^ This domain catalyzes the hydrolytic degradation of F2,6P2 into F6P and inorganic phosphate (Pi). The N-terminal domain is responsible for the synthesis of F2,6P2 from F6P and ATP (Figure 1b).^14,16,17^ The PFKFB3 protein is ubiquitously expressed, with especially high levels in proliferating tissues, transformed cells, solid tumors and leukemia cells.^18^ PFKFB3 expression could be upregulated in response to mitogenic, inflammatory and hypoxia stimuli and during the DNA synthesis phase of the cell cycle.^18^ Considering its significance in cancer metabolism, further explanation of the function of PFKFB3 in diverse cancers is necessary.

## Regulatory mechanisms of PFKFB3

**Figure 2:**
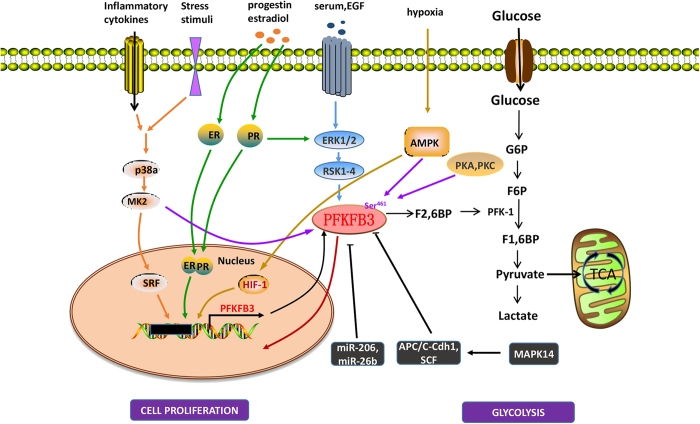
Signaling pathways involving PFKFB3. Numerous molecules are associated with PFKFB3 regulation. (1) Progestin, estradiol and hypoxia induce binding of the transcription factors PR, ER and HIF, respectively, to their responsive elements in the PFKFB3 promoter. Inflammatory cytokines and stress stimuli increase PFKFB3 production via the P38/MK2/SRF pathway. Serum and EGF function through the ERK1/2 (extracellular-signal-regulated kinase)/RSK1–4 (ribosomal S6 kinase) pathway, and progestin also regulates glycolysis through this pathway as a secondary mechanism. (2) MiR-206 and miR-26b inhibit PFKFB3 by interacting with 3′UTR of PFKFB3 mRNA. Other negative regulators of PFKFB3, such as ubiquitin ligase APC/C-Cdh1 and SCF, catalyze the degradation of the PFKFB3 protein, which in turn results in decreased glycolysis in cells. (3) PFKFB3 is phosphorylated at Ser^461^ within the C-terminal region by MK234, AMPK38, PKA and PKC.

The oncogenic Ras signaling pathway has been invoked as a central regulator of the glucose metabolism of cancer via the activity of PFKFB3.^19,20^ Ras inhibition in glioblastoma downregulates hypoxia-inducible factor-1 alpha (HIF-1α), reducing the expression of the *pfkfb3* gene and causing glycolysis shutdown and cell death.^21^ Constitutive HER2 expression increases PFKFB3 expression and glucose metabolism in breast cancer cells.^22^ Loss of p53 and PTEN and/or other tumor suppressor functions also stimulates glycolysis in part by activating the regulatory bifunctional PFKFB3 family.^23,24^ In addition, the transcriptional co-repressor myeloid translocation gene 16 (MTG16) could act as a brake on glycolysis, stimulating mitochondrial respiration and inhibiting cell proliferation through suppression of PFKFB3–4.^25^ Different stimuli have been reported to induce gene expression of pfkfb3. For example, hypoxia,^26^ progestin^27^ and estradiol^28^ induce PFKFB3 expression through interactions of HIF-1, progesterone receptor (PR), and estrogen receptor (ER) with their own consensus response elements located at the pfkfb3 promoter. Circadian-driven transcription factor ‘CLOCK’ could also bind to pfkfb3 promoter at ‘E-box’ site to increase the transcription of pfkfb3 in cancer cells. PFKFB3 inhibition significantly retarded the growth of implanted human tongue cancer cell in mice only at certain time points within the circadian cycle. This finding indicates the significance of time-based PFKFB3 inhibition in cancer treatment.^29^ Growth factors, such as insulin,^30^ pro-inflammatory molecules such as interleukin 6 (IL-6),^31^ lipopolysaccharide (LPS) and adenosine^32^ or different stress stimuli (NaCl, H_2_O_2_, UV radiation or anisomycin)^33^, increase *pfkfb3* gene expression levels. The major signal pathways involved in PFKFB3 regulation are shown in Figure 2.

The mRNAs of all PFKFB3 isoforms contain multiple copies of the AUUUA instability motif in their 3′UTR AU-rich elements.^34,35^ It was reported recently that miR-206 and miR-26b directly interact with the 3′UTR of PFKFB3 mRNA, resulting in attenuation of glycolysis in breast cancer and osteosarcoma, respectively.^36,37^ Other miRNAs, including hsa-miR-26b-5p and hsa-miR-330-3p, are also expected to have binding sites in the 3′UTR of PFKFB3, although functional validation remains to be performed.^37^ The PFKFB3 isoenzyme is phosphorylated at a consensus site, Ser^461^, within the C-terminal region by mitogen-activated protein kinase-activated protein kinase 2 (MK2),^33^ AMP-activated protein kinase (AMPK),^38^ protein kinase A (PKA) and protein kinase C (PKC),^39^ thus making it responsive to multiple external signals. S-glutathionylation^40^ and demethylation^41^ of PFKFB3 induced by high reactive oxygen species (ROS) in cancer cells cause a shift of glucose utilization from glycolysis toward the NADPH-producing pentose phosphate pathway (PPP), resulting in ROS detoxification. The PFKFB3 isoenzyme is degraded by the E3 ubiquitin ligase anaphase-promoting complex/cyclosome-cadherin1 (APC/C-Cdh1) via the KEN box.^42^ A decrease in the activity of APC/C-Cdh1 in mid-to-late G1 phase leads to the accumulation of PFKFB3.^42^ PFKFB3 is also a substrate for another ubiquitin ligase, SKP1-CUL1-F-box-protein (SCF), at the onset of S-phase via the DSG box, and thus the activity of PFKFB3 occurs in a short interval, coinciding with a peak in glycolysis in mid-to-late G1.^43^ The findings led to the identification of the roles of these ubiquitin ligases in the metabolic regulation of the cell cycle and, consequently, cell proliferation. The degradation of PFKFB3 by those two enzymes induced by mitogen-activated protein kinase 14 (MAPK14) also leads to the reduction of ROS.^44^ The detoxification of ROS related to PFKFB3 is presented in Supplementary Figure 1. PFKFB3 is differentially regulated both at the transcriptional and post-transcriptional levels (Table 2).

## Roles of PFKFB3 beyond glycolysis

Although the glycolytic role of PFKFB3 in cancer progression has been the subject of numerous functional studies, some researchers have also focused on the functions of PFKFB3 beyond glycolysis. Recent observations have established that PFKFB3 is also trafficked to the nucleus in multiple cell lines via a highly conserved nuclear localization motif in the C-terminal domain, and ectopic expression of wild-type PFKFB3 in the nucleus stimulates cellular proliferation without an effect on glucose metabolism.^45^ The product of PFKFB3, F2,6P2, activates cyclin-dependent kinases (Cdks) and then stimulates the Cdk-mediated phosphorylation of the Cip/Kip protein p27, which in turn results in decreased levels of p27 due to ubiquitination and proteasomal degradation by Cdk1.^45^ As p27 is a potent suppressor of the G1/S transition and activator of apoptosis, the known requirement for PFKFB3 for cell cycle progression and prevention of apoptosis may be partly due to the ability of F2,6P2-induced p27 degradation.^46,47^ The effect of siRNA silencing of PFKFB3 is reversed by co-siRNA silencing of p27.^47^ These results confirm that PFKFB3 expression may not only be essential for the regulation of glycolysis in the cytoplasm, but also in the control of the cell cycle in the nucleus and maintenance of an anti-apoptotic state.

## Roles of PFKFB3 in cancer

**Figure 3:**
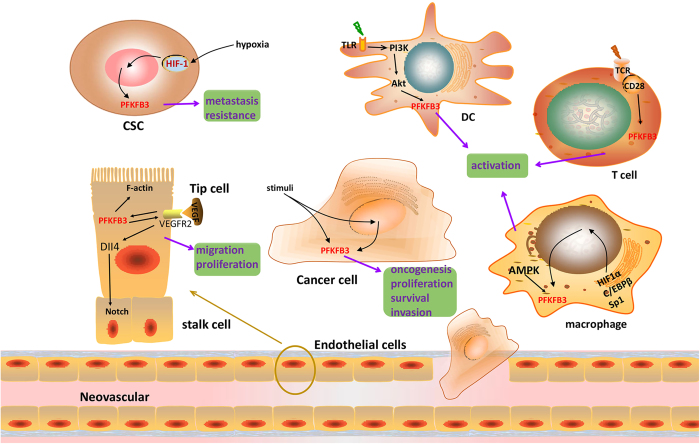
Roles of PFKFB3 in different cancer cells. High levels of the PFKFB3 isoenzyme have been proven to promote the oncogenesis, proliferation and survival of cancer cells. Elevated PFKFB3 in CSCs has been estimated to be related to distant metastasis and poor clinical outcome. PFKFB3 is apparently induced by hypoxia in CSCs. Silencing of PFKFB3 impairs vessel sprouting due to defects in both migrating tip and proliferating stalk cells. PFKFB3, compartmentalized with F-actin in lamellipodia, provide ATPs for vascular sprouting, and VEGFR2 induces PFKFB3 and activates Notch signaling. Immune cells shift from OXPHO to glycolysis when activated. The TLR/PI3K/Akt signaling pathway controls this shift in DC cells. In T cells, PFKFB3 is induced downstream by the TCR/CD28 receptor. PFKFB3 expression is increased by the transcription factors HIF1α, C/EBPβ and Sp1 in macrophages, and the PFKFB3 enzyme is phosphorylated by AMPK.

Loiseau *et al.*^48^ discovered that there was no bisphosphatase activity of PFKFB in hepatocellular carcinoma (HCC) cells, which was hypothesized as a key mechanism accounting for the loss of control of glycolysis. Although multiple PFKFB isoforms are almost certainly co-expressed in these tumor cells, the absence of bisphosphatase activity supports the explanation that the dominantly expressed PFKFB enzyme in these cells is PFKFB3. PFKFB3 has been suggested to play a crucial role in many types of tumor cells as well as various cells in the tumor microenvironment. The following sections and Figure 3 summarize recent advances of PFKFB3 in different tumor cells, tumor stem cells and tumor environment cells.

### Targeting PFKFB3 in cancer cells

Enhanced glycolysis is important for cancer development.^51^ As a key regulator of glycolysis, PFKFB3 plays an important part in oncogenesis and the survival and proliferation of cancer cells in the tumor microenvironment. The roles of PFKFB3 in different cancer cell lines and possible mechanisms are summarized in Table 3. PFKFB3 has been studied in various cancer cells. Most studies have demonstrated that cancer cell growth, proliferation, migration and metastasis are promoted when PFKFB3 expression is increased or the PFKFB3 isoenzyme is phosphorylated. In addition, gene expression inhibition by siRNA in HeLa cells^47,52^ and colon carcinoma cells,^53^ or by miRNA in breast cancer cells^36^ and osteosarcoma cells^37,54^ decreases the growth, proliferation and migration of these cells. PFKFB3 inhibitor 3-(3-pyridinyl)-1-(4-pyridinyl)-2-propen-1-one (3PO) and its derivation 1-(4-pyridinyl)-3-(2-quinolinyl)-2-propen-1-one (PFK15) have been shown to reduce glucose metabolism and exhibit potent antitumor activity in several human cancer xenograft models, including tongue carcinoma, gastric cancer and head and neck squamous cell carcinoma.^29,55,56^ These studies further proved the proto-oncogenic role of PFKFB3. A recent *in vitro* study found that PFKFB3 is a key effector protein of transforming growth factor β1 (TGFβ1), which is an inducer of epithelial–mesenchymaltransition (EMT) in tumor cells,^57^ further suggesting a role of PFKFB3 in the cancer invasion process. Most of the literature has reported that increased PFKFB3 promotes tumorigenesis and proliferation. However, experimental result in astrocytoma cells is inconsistent. Zscharnack *et al.*^58^ found that the PFKFB3 splice variant UBI2K4 is downregulated in high-grade astrocytoma relative to low-grade astrocytomas and corresponding non-neoplastic brain tissue. Overexpression of UBI2K4 decreased cell viability and anchorage-independent growth of U87 cells. Consequently, further study is needed to elaborate the exact role of PFKFB3 in different cancer cells.

Under different stimuli, the mechanisms involved in PFKFB3 regulation in different cancer cell lines differ. Even under the same stimuli, cancer cells also display diverse regulatory mechanisms for PFKFB3. For instance, under progestin stimulation, dual mechanisms operate to ensure glycolysis in breast cancer cells (increased expression and increased phosphorylation of PFKFB3).^27^ To protect cancer cells from harm from high ROS, HeLa cells and human leukemia U937 cells utilize two different modifications of PFKFB3 (S-glutathionylation and demethylation of PFKFB3).^40,41^ Desideri *et al.*^44^ suggested that decreased PFKFB3 entails loss of autophagy in HeLa cells, leading to increased resistance to nutrient deprivation. The possible mechanism is that ROS, a mild activator of autophagy, is detoxified by decreased PFKFB3 by shifting glycolysis to PPP. However, another study reported an opposite outcome that the selective inhibition of PFKFB3 induces increased autophagy in HCT-116 colon adenocarcinoma cells as a survival mechanism.^53^

Together, the roles of PFKFB3 on cancer cells are widely studied in cell lines and xenograft models. However, its functions and mechanisms are not exactly the same. A genetically engineered cancer *in vivo* model is still lacking for further investigation.

### Targeting PFKFB3 in cancer stem cells (CSCs)

CSCs are a subgroup of cells within a tumor that have the ability of (1) self-renewal and differentiation into multiple cell types when transplanted;^59^ (2) initiating new tumors or responsible for the dissemination of metastases;^60^ (3) resisting to anticancer drugs or radiations.^61,62^ Pacini *et al.*^63^ suggest that the undifferentiated state of stem cells is characterized by a decrease in oxidative phosphorylation, a reduced level of intracellular ATP and a smaller production of ROS. CSCs from colon carcinoma,^64^ osteosarcoma,^65^ epithelial ovarian cancer^66,67^ and breast cancer^68^ were proved to rely on the glycolysis pathway rather than oxidative phosphorylation (OXPHOS) for their energy needs. However, leukemia^69^ and pancreatic adenocarcinoma^70^ support OXPHOS as the primary energy source of CSCs. Even in the same type of tumor CSCs, the glucose metabolic patterns from different studies differ.^71,72^ Nevertheless, the peculiar metabolic characteristic of CSCs becomes a therapeutic and diagnostic opportunity in cancer research.

CD44^+^CD24^−^ breast cancer stem-like cells are enriched in tumor-initiating and chemotherapy-resistant cells.^73,74^ *Pfkfb3* is one of the genes in the CD44^+^CD24^−^ cell gene signature that has been related to an enhanced risk of distant metastasis and poor clinical outcome in breast cancer patients.^86,87^ Cieslar-Pobuda *et al.*^88^ revealed that breast CSCs can be distinguished from induced pluripotent stem cells (iPS) or surrounding breast cancer cells based on differences in PFKFB3 and PFK-1 expression. They found that PFKFB3 and PFK-1 expression are higher in CSCs than in iPS cells. When cultured under hypoxic conditions, iPS cells and cancer cells change the expression levels of PFKFB3 and PFK-1 similarly in CSCs, supposing that CSCs might enhance glycolysis due to hypoxia-mediated modulation of restriction point (R-point) markers such as PFK-1 and PFKFB3. The different expression levels of PFKFB3 and PFK-1 among CSC, iPS cells and non-stem cancer cells suggest improved prospects for the more precise detection of CSCs and for clinical applications of stem cell-based therapies.

### Targeting PFKFB3 in endothelial cells (ECs)

The blood vessel lumen is lined by a monolayer of ECs including tip cells and stalk cells, and each performs specific functions.^89,90^ ECs are also glucose addicted and highly glycolytic even in the presence of ample oxygen.^91,92^ A few possible explanations are as follows: (1) glycolysis generates ATP more rapidly to meet energy needs for EC motility compared to oxidative metabolism, thus quickly restoring more oxygen supply to the perivascular tissue; (2) glycolysis side pathways produce macromolecules needed for biomass duplication during cell division, which may further contribute to rapid vascular sprouting; (3) ECs can still depend on glycolysis to sprout in the milieu with less oxygen and glucose because glucose diffuses further away from vessels than oxygen; (4) by primarily maintaining glycolysis metabolism, ECs minimize the production of ROS, protecting ECs from their hyperoxic stress microenvironment.^91,93–95^

Tumor angiogenesis, represents one of the central hallmarks of cancer, is the growth of new blood vessels which supply nutrients for tumor growth, expansion and progression.^96^ High microvessel density in tumor specimen correlates metastasis, recurrence, poor prognosis in many malignancies.^97,98^ During angiogenesis, endothelial cells proliferate so that new capillary blood vessels can develop from preexisting microvessels to tolerate blood flow. Induction of PFKFB3 by vascular endothelial growth factor (VEGF) promotes angiogenesis and endothelial migration by regulating the tube formation of filopodia and lamellipodia and directional migration. Silencing of PFKFB3 in ECs reduces vascular sprouting by decreasing the migration of tip cells and proliferation of stalk cells.^89,91,99,100^ A few other mechanisms may also underlie this effect: (1) PFKFB3 compartmentalizes with F-actin in lamellipodia to create an assembly line of glycolysis, facilitating efficient and rapid local ATP production to fuel migration;^91^ (2) the increase in lactate upon increased PFKFB3 stimulates angiogenesis via activation of HIF-1α and upregulation of VEGF receptor 2 (VEGFR2);^101^ (3) PFKFB3 also control cell proliferation via glycolysis-independent Cdks activities in the nucleus.^45^

In contrast to traditional anti-angiogenic therapy that aims to inhibit angiogenesis, an emerging paradigm is to normalize characteristically chaotic tumor vasculature in order to improve blood perfusion, which could decrease hypoxia and increase drug accessibility. The normalized vessels might also resist shedding of cancer cells from the primary tumor, potentially reducing tumor metastasis.^102,103^ A recent study found that inhibition of PFKFB3 in melanoma tumor endothelial cells (TECs) induces tumor vessel normalization, thus reducing cancer cell invasion, intravasation and dissemination, and contributing to increased response to chemotherapy.^104^ This effect is probably achieved by reducing VE-cadherin endocytosis and inflammation in ECs and making pericytes more quiescent and adhesive under reduced glycolysis. By decreasing NF-κB signaling, TECs also lower the expression of cancer cell adhesion molecules, which contributes to metastasis inhibition of cancer cells.^104^ Thus, targeting EC metabolism through PFKFB3 might offer unprecedented opportunities for anti-angiogenic therapies and inhibition of tumor growth.

### Targeting PFKFB3 in immune cells

The Warburg effect has also recently been associated with many immune cell activities. For example, resting or quiescent T cells will switch from OXPHOS to aerobic glycolysis to provide sufficient ATP and precursor molecules for proliferation and survival when encountering antigens.^105–107^ Chang *et al.*^108^ found that when activated T cells are provided with co-stimulation and growth factor stimulation but are blocked from engaging in glycolysis, their ability to produce interferon-γ (IFN-γ) is markedly compromised. IFN-γ is a cytokine contributing to the inhibition of the development of chemically or virus-induced tumors.^109^ T-cell activation is associated with a rapid increase in intracellular PFKFB3. PFKFB3 may be the dominant PFKFB family member involved in TCR/CD28-induced F2,6P2 synthesis and glycolysis.^110^ 3PO, an inhibitor of PFKFB3, can suppress T-cell-dependent immunity and induce apoptosis of T cells *in vitro* and *in vivo.*^110^ Researches on the roles of PFKFB3 in the antitumor immunity associated with T cells mediated are still rare except the valuable work done by Chesney. They found a PFKFB3 inhibitor, PFK-158, could decrease tumor-infiltrating Th17 cells and myeloid-derived suppressor cells, and increase tumor-infiltrating CD4^+^ and CD8^+^ T cells in the tumors of B16-F10 melanoma-bearing mice.^111–113^ However, the functions of different immune cells are different and complicated. Thus, a clarification of the function of PFKFB3 in different immune cells, immune responses and immune stages is necessary for a deeper understanding of the role of PFKFB3 in antitumor immunity.

Augmented aerobic glycolysis is also important for regulating the activation and function of dendritic cells (DC) and macrophages.^32,114,115^ DC activation and maturation are induced by Toll-like receptors (TLRs) ligands, stimulating a profound metabolic transition to aerobic glycolysis. The phosphatidylinositol 3ʹ-kinase (PI3K)/Akt signaling pathway controls this metabolic switch.^116^ Increased PFKFB3 expression could be essential in protecting the viability of macrophages to develop their long-term defense and reparative functions in the inflammatory microenvironment. The expression of the transcription factors HIF1α, C/EBPβ, and Sp1 is activated in macrophages treated with LPS. LPS also increases AMPK activity in macrophages, which increases PFKFB3 phosphorylation.^32^ Because these immune cell experiments were all performed *in vitro* and the cancer microenvironment *in vivo* is completely different, more studies *in vivo* and in cancer models are needed to illustrate the function of glycolysis in immune cells within cancer.

Chronic inflammation is acknowledged as the cause of various human cancers.^117,118^ For example, patients with inflammatory bowel disease have an increased risk for colorectal cancer (CRC).^119,120^ The AUUUA instability element found in the mRNA of pfkfb3 is also found in several inflammatory cytokine mRNAs (for example, IL-1, IFN-γ and granulocyte/macrophage colony-stimulating factor, GM-CSF).^34,35,121^ The inflammatory cytokine IL-6 stimulates aerobic glycolysis and promotes cell proliferation and migration in CRC cells. *Pfkfb3* was the gene most downregulated by an anti-IL-6 receptor antibody in colorectal adenoma tissues.^77^ We therefore speculate that the tumor inflammatory environment could be alleviated through PFKFB3 inhibition.

## Therapeutic potential of PFKFB3 inhibitors in the treatment of cancer

As knowledge of PFKFB3 in cancer metabolism accumulates, there has been an increased interest in the identification and development of PFKFB3 inhibitors. It is known that current standard chemotherapeutic and irradiation protocols mainly target rapidly dividing cells. The data from Liu *et al.*^122^ suggest the efficacy of these treatments could be enhanced by inhibition of glycolysis aimed specifically at slower growing cancer cells.

3PO is a well-studied suppressor of the basal catalytic activity of the PFKFB3 isozyme. It decreases intracellular F2,6P2, thus suppressing glycolytic flux in transformed cells. 3PO functions through its binding to the sites that the PFKFB3 protein binds and functions. Consequently, binding competition between 3PO and F6P for the binding area is possible.^84^ However, Lineweaver-Burk double-reciprocal plot analyses have shown that 3PO exhibits a complex mechanism in the inhibition of PFK-2 activity that is both competitive and uncompetitive. Introduction of 3PO to various cancer cells, such as breast cancer,^123^ bladder carcinoma cancer^83^ and hepatocellular carcinoma^84^ induces cytotoxicity, apoptosis and growth inhibition. An advantage is that 3PO and its optimized derivatives do not affect serum glucose, red blood cell and white blood cell concentrations when administered daily *in vivo.*^110^

PFK15, a potent derivation of 3PO, was recently demonstrated^55,56^ to (1) cause cell cycle arrest in G0/G1 phase by blocking the cyclin-CDKs/Rb/E2F signaling pathway; (2) induce apoptosis through mitochondria; (3) inhibit invasion by downregulating focal adhesion kinase (FAK) and upregulating E-cadherin. Additionally, compared with other PFKFB3 inhibitors, PFK15 displays potent and selective activity against PFKFB3 with low cytotoxicity.^55,124^ The improved apoptosis potency of PFK15 was greater than the metabolic changes it induced.^124^ Another potent and selective inhibitor of PFKFB3, PFK-158, displays broad antitumor activity and immunomodulatory effects in multiple human and syngeneic preclinical models.^111–113^ A phase I clinical trial demonstrated that PFK-158 was successfully completed in July 2016. PFK-158 presented safety and anticancer activity in 6 of 19 evaluable patients with various advanced solid tumors (http://www. advancedcancertherapeutics.com). Other inhibitors of PFKFB3 have also been identified, such as 5-triazolo-2-arylpyridazinone,^125^ 1-(3-pyridinyl)-3-(2-quinolinyl)-2-propen-1-one (PQP),^126^ 5, 6, 7, 8-tetrahydroxy-2-(4-hydroxyphenyl) chrome-4-one (N4A) and 7, 8-dihydroxy-3-(4-hydroxyphenyl) chromen-4-one (YN1).^127^ The further identification of small-molecule inhibitors of PFKFB3 may provide a new avenue for the development of novel chemotherapeutic agents.

## Conclusion and perspectives

Knowledge of the function of PFKFB3 in cancer has advanced considerably in the past several years. In this review, we summarized the function of PFKFB3 in tumor metabolism and elucidated the regulatory mechanisms of PFKFB3. Furthermore, the role of PFKFB3 in human tumor cells, CSCs, ECs and immune cells, was discussed in this review. PFKFB3 represents a promising target for tumor treatment. However, until now, no PFKFB3 inhibitors have been approved to treat patients with cancer. 3PO is a potent inhibitor of PFKFB3 but poor water solubility makes this compound clinically unavailable. Other potent and selective inhibitors of PFKFB3, such as PFK15 and PFK-158, are under clinical trials for treating late-stage cancer patients. Recently emerged nanotechnology-based drug delivery carriers^128–130^ have the ability to formulate various hydrophobic anticancer agents including 3PO,^131^ thereby showing the potentials to improve the anticancer efficacy when used *in vivo*. On the other hand, specifying resistance mechanisms triggered by targeted therapies would allow for the specific selection of drug combinations.

It was recently demonstrated that lactic acidosis arising as a result of tumor metabolism allows cancer cells to develop strong resistance to glucose deprivation-induced cell death.^132^ The theory that changing the pH of the tumor microenvironment with bicarbonate has been successfully applied in clinical treatment for patients with HCC that is not amenable to surgery,^133^ which provided new insights into treating tumors by targeting their metabolism and microenvironment. Reasonably, we believe combining PFKFB3 inhibitor with environmental cells inhibition, such as immune suppressors or angiogenesis inhibitors, would probably generate a better effect of tumor eradication. For example, the PFKFB3 inhibitor PFK-158 could improve the antitumor activity of the immune checkpoint inhibitor anti-CTLA4 in the B16 mouse model.^112^ Furthermore, tumors could rely on other metabolic pathways for energy supply besides glycolysis. For example, increased cholesterol synthesis and steroidogenesis occur throughout prostate cancer carcinogenesis rather than the classic ‘glycolytic switch’ observed in the majority of other solid tumors.^134,135^ Increased fatty acid oxidation is sufficient for cell survival and to protect cells from glucose withdrawal-induced death in Akt-overexpressing glioblastoma.^136^ A synergistic anti-neoplastic effect of anti-metabolism agents is anticipated when combined with chemotherapeutic drugs. How these drugs interact and produce optimal effects warrants further investigations.

## Figures and Tables

**Table 1:** Comparison of the nucleotide sequences and body localization of six ubiquitous PFKFB3 isoforms

*Splice variants*	*Alias*	*Variable exons*	*Location*
UBI2K1		A, B, C, G	Low levels in the brain
UBI2K2		A, C, D, E, G	Low levels in the brain
UBI2K3		A, C, D, F, G	Brain-specific
UBI2K4	Inducible PFK-2	A, C, D, G	Preferentially expressed in human skeletal muscle
UBI2K5	Placenta PFK-2/FBPase-2 ubiquitous PFK-2/FBPase-2	A, C, G	Brain, liver, skeletal muscle
UBI2K6		A, G	Brain, liver, skeletal muscle

**Table 2:** Regulatory mechanisms of PFKFB3

*Regulator*	*Action location of PFKFB3*	*Effect on PFKFB3*	*Ref*.
Ras		Increased expression	^19,21^
HER2		Increased expression	^22^
P53		Decreased expression	^23^
PTEN	KEN box	Indirect degradation	^24^
MTG16		Decreased expression	^25^
HIF-1	HIF-1 response elements (HRE)/Ser461	Increased expression Phosphorylation	^26,38^
PR	PR response elements (PRE)/Ser461	Increased expression Phosphorylation	^27^
ER	ER response elements (ERE)	Increased expression	^28^
CLOCK	E-box	Increased expression	^29^
Stress stimuli (NaCl, H_2_O_2_, UV radiation, anisomycin)	Serum response element (SRE)/Ser461	Increased expression Phosphorylation	^33^
Insulin	Sterol regulatory elements (SRE) and E-boxes	Increased expression	^30,49,50^
Pro-inflammatory molecules (IL-6, LPS and adenosine)	Promoter	Increased expression	^31,32^
microRNA (miR-206, miR-26b, hsa-miR-26b-5p, hsa-miR-330-3p)	3′UTR	Decreased expression	^36,37^
MK2, AMPK, PKA, PKC, MAPK	Ser461	Phosphorylation	^33,38,39^
ROS	C206 at the N terminus	S-glutathionylation	^40^
	R131/R134 at the N terminus	Demethylation	^41^
APC/C-Cdh1	KEN box	Degradation	^42^
SCF	DSG box	Degradation	^43^

**Table 3:** PFKFB3 in different cancer cell lines

*Cancer cell type*	*Upstream effector*	*Possible mechanism*	*PFKFB3 expression level*	*Effect on cancer cell*	*Ref*.
Breast cancer cells	Estradiol	Increased transcription of PFKFB3 by ER	↑	Survival, growth and metastases	^28^
	Progestins	Increased transcription of PFKFB3 by PR ERK/RSK phosphorylation of PFKFB3	↑	Proliferation	^27^
	AMPK	Phosphorylation of PFKFB3	—	Mitotic arrest survival	^75^
	miR-206	Interaction with 3′UTR in PFKFB3 mRNA level	↓	Proliferation and migration inhibition	^36^
Hela cells	stress stimuli	p38/MK2 pathway	↑	Adaptation to microenvironmental conditions	^33^
	high ROS	S-glutathionylation of PFKFB3	—	ROS detoxification; cell survival and proliferation	^40^
	MAPK14	Increased degradation of PFKFB3 by APC/C-Cdh1 and SCF	↓	Increased resistance to nutrient deprivation	^44^
	siRNA	Silencing of *PFKFB3* gene	↓	Cell cycle arrest at G1/S; Increased apoptosis; anchorage-independent growth	^47,52^
Pancreatic and gastric cancer cells	hypoxia	Increased transcription of PFKFB3 by HIF-1	↑	Proliferation and survival	^76^
Colorectal cancer	IL-6	—	↑	Proliferation and migration	^77^
Colon carcinoma cell lines		Phosphorylation PFKFB3	—	Cell proliferation	^78^
	insulin	Increased transcription of PFKFB3	↑		^30^
	siRNA	silencing of *PFKFB3* gene	↓	Apoptosis	^53^
Lung adenocarcinoma cells	siRNA	Silencing of *PFKFB3* gene	↓	Decreased growth	^19^
Renal cancer cells	rasfonin	—	↑	Autophagy and apoptosis	^79^
Myeloid lineage cells	JAK2/STAT5	Increased transcription of PFKFB3 by STAT5	↑	Increased Growth	^80^
Leukemia U937 cells	high ROS	Reduced methylation of PFKFB3	—	Survive from oxidative stress	^41^
Acute myeloid leukemia cell lines	siRNA	Silencing of *PFKFB3* gene	↓	Cell proliferation inhibition; apoptosis induction	^81^
DB-1 melanoma cells	low pH exposure	Phosphorylation PFKFB3	—	Tumorigenesis and treatment resistance	^82^
Osteosarcoma cells	miR-26b	Interaction with 3′UTR in PFKFB3 mRNA level	↓	Proliferation, migration, invasion inhibition; apoptosis induction	^37,54^
Bladder cancer cell lines	3PO	Inhibition of PFKFB3	—	Reduced growth	^83^
Malignant hematopoietic and adenocarcinoma cell lines	3PO	Inhibition of PFKFB3	—	Reduced growth	^84^
Tongue cancer	3PO	Inhibition of PFKFB3	—	Proliferation inhibition, apoptosis	^29^
Pancreas cancer	TGFβ1	Increased transcription of PFKFB3	↑	Invasion	^57^
Gastric cancer cells	siRNA	Silencing of *PFKFB3* gene	↓	Proliferation and migration inhibition	^85^
	PFK15	Inhibition of PFKFB3	—	Cell cycle arrest; apoptosis; invasion inhibition	^55^
Head and neck squamous cell carcinoma cell lines	PFK15	Inhibition of PFKFB3	—	Proliferation suppression; halted cell cycle progression; induced cell apoptosis	^56^
Glioblastoma cells		—	↑(Induced)	Decreased growth rate, cell viability, anchorage-independent growth	^10,58^
