# Supplementary material for: Roles of PFKFB3 in cancer
Source: Signal Transduct Target Ther. 2017 Nov 24;2:17044–. doi: 10.1038/sigtrans.2017.44 (PMC5701083; doi:10.1038/sigtrans.2017.44)
Supplement: Supplementary Figure 1 [file sigtrans201744-s1.docx]

**
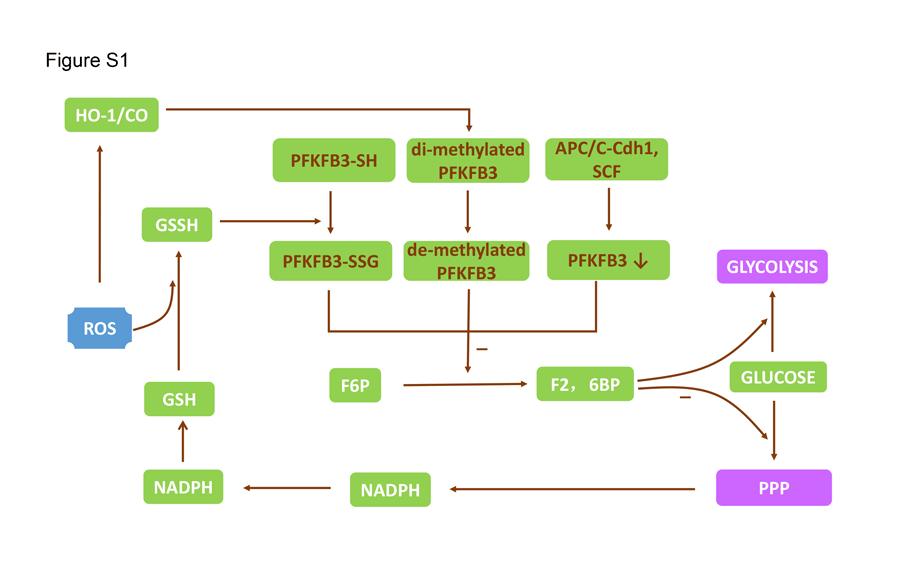
**

**Supplementary Figure 1.** Schematic illustration of ROS detoxification in cancer cells via PFKFB3 alteration. Two modifications of PFKFB3 and a decrease in PFKFB3 flux are involved in ROS regulation. ROS oxidizes reduced state glutathione (GSH) to glutathione disulfide (GSSG) in cells. S-Glutathionylation (PFKB3-SSG) of unglutathionylated PFKFB3 (PFKFB3-SH), demethylation of dimethylated PFKFB3 and decreased PFKFB3 degradation by the ubiquitin enzymes APC/C-Cdh1 and SFC all lead to decreases in F2,6BP levels and in carbohydrate flux to glycolysis. These decreases, in turn, increase the flux to PPP and the level of GSH, which protects cells from damage from ROS and maintains oxidative stress homeostasis.
